# Editorial: Use of 3D Models in Drug Development and Precision Medicine - Advances and Outlook

**DOI:** 10.3389/fbioe.2021.658941

**Published:** 2021-03-03

**Authors:** Adriele Prina-Mello, Luigi Bonacina, Davide Staedler, Dania Movia

**Affiliations:** ^1^Laboratory for Biological Characterisation of Advanced Materials (LBCAM), Department of Clinical Medicine, Trinity College Dublin, Trinity Translational Medicine Institute, Dublin, Ireland; ^2^Advanced Materials and BioEngineering Research (AMBER) Centre, Centre for Research on Adaptive Nanostructures and Nanodevices (CRANN) Institute, Trinity College Dublin, Dublin, Ireland; ^3^Department of Applied Physics, Université de Genève, Genève, Switzerland; ^4^Department of Biomedical Sciences, University of Lausanne, Lausanne, Switzerland

**Keywords:** organ-on-chip, mini-organ, 3D culture, scaffold-based cell culture, organoid, drug discovery and development

Three-dimensional (3D) *in vitro* models in the drug development pipeline can help selecting the most promising and safe drug candidates at the pre-clinical stage, prior to clinical trials, reducing and sometimes even replacing animal studies in accordance with the “3Rs (Reduction, Refinement and Replacement) principle” (Herrmann and Jayne, 2019). Several types of 3D *in vitro* cultures have been developed for this purpose, including advanced models such as organ-on-chips and microfluidic models (Sontheimer-Phelps et al., 2019; Peck et al., 2020), organoids (Kim et al., 2020), and mini-organs (Lawlor et al., 2020). These models have also opened many new opportunities and research directions in the drug discovery space. For example, 3D organoids generated from cells harvested from patients can be applied toward a personalized medicine approach. Moreover, the development and translational investigation of new therapies or treatments for degenerative or regenerative applications, can be expedited by tissue engineering solutions powered by the current knowledge in 3D *in vitro* modeling. This facilitates drug formulation and screening with a direct input into the regulatory science and industrial technological innovation pipeline.

This Research Topic covers the areas of the development, use and validation of *in vitro* 3D models where novel methodologies and findings demonstrate the key role of three-dimensionality in biology, and provide a platform to increase the success rate in translating new diagnostic and treatment solutions into real clinical innovative approaches to the benefit of patients. This Research Topic features five review and perspective articles, which elucidate the multiple facets of the field of alternative models in drug discovery and provide critical considerations for its short- and long-term development. These reviews are complemented by three original research articles, which help contextualizing the challenges and the potential of the state-of-the-art in 3D *in vitro* modeling.

In the cancer research area, Kitaeva et al. contributed with a review on advanced *in vitro* models. This manuscript provides an in-depth comparison of different methodologies including two- and three-dimensional cultures, Boyden chambers, microfluidic systems, and 3D bioprinting. Mondadori et al. performed a systematic literature review updated to January 2020 on the microfluidic models available for the study of cancer and immune cells extravasation highlighting the key role of biophysical, biochemical, and environmental factors in the several studies analyzed. Similarly, Bracher et al. discuss the need for a systematic approach to review *in vitro* methods in brain tumor research. This approach would enable to identify relevant appraisal criteria to aid planning and/or evaluation of brain tumor studies using advanced *in vitro* methods.

In the tissue engineering field, the review by Thompson et al. provides insights on commercially available organ-on-chip platforms incorporating active biomechanical stimulation. The authors highlight instances where mechanical stimuli can drastically alter a given biochemical response with relevance to pre-clinical studies. They also critically discuss which level of approximation of the *in vivo* conditions is sufficient for the proposed screening applications. In their review entitled “Building Scaffolds for Tubular Tissue Engineering,” Boys et al. from University of Cambridge, discuss some of state-of-the-art methods for producing hollow and tubular systems. The latter deem essential to provide crucial tissue structures including vasculature, the intestines, and the trachea. The authors carefully review different methodologies such as casting, electrospinning, rolling, 3D printing, and decellularization.

In the lung research area, Movia et al. present a perspective article on the status and the outlook of *in vitro* respiratory models for toxicity studies. Notably this contribution provides a compendium of regulatory information useful for all researchers in the field. Ramos-Gomes et al. took a distinct perspective for their contribution to the topic, by introducing a novel method to study nanoparticle-cell interactions in the lung. This work details a clever imaging protocol to obtain time-series at high spatial resolution in an *ex vivo* system. The authors discuss the potential of this approach for assessing novel therapeutic strategies with a special emphasis on nanomedicine.

In the organoids and mini-organs field, Zietek et al. describe the applicability of 3D organoids for *in vitro* investigation of intestinal biochemical processes related to transport and metabolism of nutrients and drugs. The authors, relying on a wide range of methodologies, provide a thorough assessment of the robustness and reliability of intestinal organoids. Whereas, Govindan et al. detail the step-by-step procedure leading to the generation of human mini-brains (i.e., 3D brain *in vitro* spheroid models comprising of neurons and glial cells, generated from human induced pluripotent neural stem cells) and the protocols to successfully label projection neurons, perform immunohistochemistry and 3D imaging at large scales.

In conclusion, this Research Topic provides an extended overview of the advanced *in vitro* approaches that can be applied to drug development and precision medicine. We are sure the reader will find this Research Topic as a useful reference for state-of-the-art in the fast-growing field of 3D cultures, organ-on-chip, organoids and mini-organs and their use in the relevant toxicology, bioengineering, and biomedical fields.

## Author Contributions

All authors contributed to the drafting and finalization of the editorial.

## Conflict of Interest

The authors declare that the research was conducted in the absence of any commercial or financial relationships that could be construed as a potential conflict of interest.
